# Turmeric-Fortified Cow and Soya Milk: Golden Milk as a Street Food to Support Consumer Health

**DOI:** 10.3390/foods11040558

**Published:** 2022-02-16

**Authors:** Folake Idowu-Adebayo, Vincenzo Fogliano, Anita Linnemann

**Affiliations:** 1 Food Quality and Design, Department of Agrotechnology and Food Sciences, Wageningen University and Research, 6708 WG Wageningen, The Netherlands; folake.idowu-adebayo@wur.nl (F.I.-A.); vincenzo.fogliano@wur.nl (V.F.); 2 Department of Food Science and Technology, Federal University Oye-Ekiti, Oye-Ekiti 371104, Nigeria

**Keywords:** sustainable meals, calcium, iron, zinc, protein, total antioxidant activity, total phenol content

## Abstract

We studied plant-based milk from soya beans as a means to release and convey the bound antioxidants in turmeric to benefit consumer health. This was compared to cow milk as a carrier because soya milk consumption as an alternative to cow milk is increasing globally. Hence, turmeric paste was added to milk to investigate the release of turmeric antioxidants when changing the matrix (cow vs. soy), the amount of turmeric paste (0%, 2%, and 6%), and the effect of heating (with and without). Proximate, physicochemical, and mineral analysis were carried out for all samples. The total phenol content (TPC) and total antioxidant activity were measured using Folin–Ciocalteu and Quencher methods. Protein ranged from 2.0% to 4.0%, and minerals ranged from 17.8 to 85.1, 0.37 to 0.53, and 0.29 to 0.30 mg/100 mL for calcium, iron, and zinc, respectively. TPC ranged from 0.01 to 0.147 GAE (g/kg) and antioxidant activity from 7.5 to 17.7 TEAC (mmol Trolox/kg sample). Overall, turmeric added nutritional and chemical value to all the samples with and without heat treatment. However, turmeric-fortified soya milk samples showed the highest protein, iron, zinc, TPC, and antioxidant activity. This study identified a cheap, additional nutrient source for developing-countries’ malnourished populations by utilizing soya bean milk to produce golden milk.

## 1. Introduction

In Nigeria, turmeric (*Curcuma longa* L.) is widely available. An increase in turmeric production and processing could improve the country’s economic development and nutritional status [1] and is, therefore, stimulated by the government. Turmeric is considered a health-supporting food ingredient, related primarily to the lipophilic bioactive compound curcumin with antioxidant properties and anti-inflammatory properties [2]. Familiar street-vended drinks (soya milk and the hibiscus–water-based drink called zobo) have been fortified with turmeric; we previously reported the consumers’ overall acceptability of these novel turmeric-fortified drinks [3]. The antioxidant and nutritional quality of street-vended zobo (*Hibiscus sabdariffa*) drink fortified with turmeric has also been reported [4]. 

Turmeric milk, called “*haldi doodh*” in India, is an example of a traditional drink often consumed to treat a sore throat and as a home therapy for fever [5]. Various ailments such as duodenal ulcer, asthma, malaria, cough, and cold can also be addressed with boiled turmeric milk [6]. Moreover, consumers’ increasing preference for natural and healthy foods has contributed to the popularity of turmeric milk, which is now known as “golden milk” globally. Since golden milk is widely consumed and its consumption is gaining popularity globally, the claims of its antioxidant and nutritional qualities must be scientifically validated.

Soya milk is a watery extract of soya beans (*Glycine max* L.). In the northern part of Nigeria, soya beans are grown and play a vital role in the dietary pattern of the population for their nutritional value. Soya bean is the plant with the highest percentage of GMOs grown worldwide. Genetically modified soya beans account for 82% of the overall crop. Soya is grown all over the world and is a nutrient-dense food that is also a good source of protein, providing vital dietary amino acids [7]. According to Mazumder and Begum [8], soya milk contains significant amounts of essential and branched-chain amino acids. The differences in amino acids between cow and soya milk are further reported in their study. The non-availability and high cost of cow milk in developing countries have led to the promotion of milk from plants, especially soya milk [9]. The commercial production of soya milk, sold as a street-vended drink in these countries, especially Nigeria, helps mitigate the vendors’ poverty [10]. Globally, the absence of gluten, lactose, and cholesterol has made soya milk a good alternative for those with lactose intolerance or galactosemia and vegetarians [11]. Hence, soya milk has also gained much popularity as a vegan and healthy drink in developed countries where lactose intolerance among the elderly and cow milk allergy among infants and children are increasing [12,13]. Additionally, soya milk is similar to cow milk in appearance, and its consistency makes it a good milk analog [14]. 

Cow milk is a source of energy, protein, and fat, and a high milk intake contributes to increased height, stronger bones, and better dental health [15]. In addition, cow milk contains all B vitamins and has antioxidant properties. Ascorbic acid is the primary water-soluble antioxidant in milk, and vitamin A and E are the primary lipid-soluble antioxidants [16]. Several studies have been carried out on both cow and soya milk’s phenolic and antioxidant activity. Temperature, pH, protein type, and concentration have been reported as fundamental parameters that can affect protein–phenolic interactions [13,17,18,19]. 

In recent years, milk products have been reported as a distinctive carrier, suitable to distribute nutrients and phytochemicals for health benefits in our nutritional system [20,21]. Investigating the use of soya milk in developing many native dairy-based foods that have demand globally has been recommended [12,22,23,24]. Cow and soya milk have been fortified with herbs, spices, and plant extracts [11,20,25,26]. Such fortification with herbs and spices or their extracts uses such fortified milk products as carriers for health-supporting compounds.

This study investigated whether turmeric-fortified soya milk is a suitable drink to improve the diet of the low-income population of urbanized areas in developing countries. We explored the differences in the interaction between turmeric components and cow milk vs. soya proteins. Hence, the effect of changing the matrix (cow vs. soya), the amount of turmeric paste (0%, 2%, and 6%), and the effect of heating (with and without) on nutritional and chemical properties of the so-called golden milk were assessed. 

## 2. Materials and Methods

### 2.1. Materials

Whole, skimmed, and soya milk produced in the Netherlands were purchased at a local supermarket (Wageningen, The Netherlands). Turmeric rhizomes were purchased from the Omuooke-Ekiti (7°45′29.99″ N, 5°43′20.17″ E) market located in Ekiti state, Nigeria. All the samples were taken to the laboratory of the Food Quality and Design group of Wageningen University and Research (Wageningen, The Netherlands) for preparation and analysis. All chemicals used were of analytical quality.

### 2.2. Preparation of Turmeric-Fortified Milk

**Figure 1:**
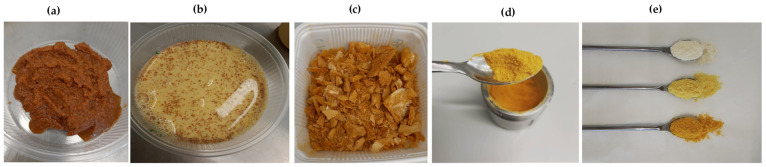
The preparation of homogeneous turmeric-fortified milk samples: (**a**) fresh turmeric paste, (**b**) 6% turmeric-fortified whole milk, (**c**) freeze-dried 6% turmeric-fortified whole milk, and (**d**) milled freeze-dried 6% turmeric-fortified whole milk (**e**) from left to right, 0%, 2%, and 6% milled freeze-dried turmeric-fortified whole milk samples.

Turmeric-fortified milk was prepared by adding two fresh turmeric paste concentrations, namely 2% and 6% turmeric in whole, skimmed, and soya milk. Milk without fresh turmeric paste (0%) was made as the control sample for all the milk types. All samples were prepared with and without heat treatment. Fresh turmeric paste was produced at a turmeric:tap water ratio of 1:2 on a weight basis by mixing with a blender (Waring Heavy-Duty Laboratory Blender, Wertheim, Germany) at high speed for 10 min until a fine paste was formed (Figure 1a).

Whole, skimmed, and soya milk were purchased in amounts of at least six liters at once for each milk type and mixed in a large bucket to minimize biological variation. Next, one liter of turmeric milk was prepared for each treatment (Figure 1b). The samples received a heat treatment by gently boiling them for 2 min on an electric cooker (Tristar KP-6245, Mechernich, Germany) to prevent the milk from curdling. Due to the nonhomogeneous distribution of particles in the turmeric-fortified milk as shown in Figure 1b for 6% turmeric-fortified whole milk, all samples were freeze-dried using a vacuum freeze dryer (Zirbus Technology GmbH, Bad Grund, Germany) for 168 h at a minimum of −20 °C (Figure 1c). After freeze-drying, the samples were milled in a planetary ball miller (Retsch MM 400, Haan, Germany) to obtain a homogeneous powder (Figure 1d) for all samples (Figure 1e).

**Figure 2:**
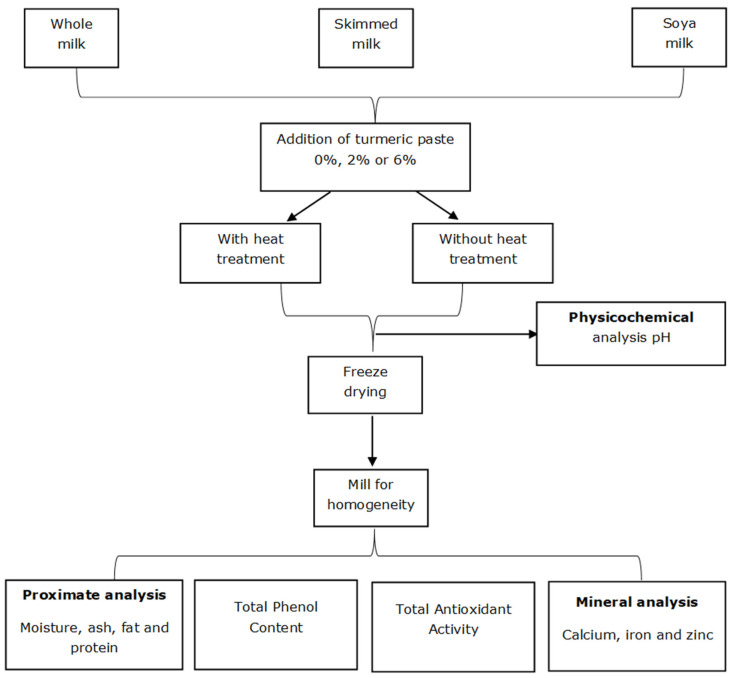
Schematic diagram of the preparation of the turmeric-fortified cow and soya milk samples with the subsequent laboratory analyses.

Figure 2 schematically shows the preparation of turmeric-fortified whole, skimmed, and soya milk samples as well as the laboratory analyses.

### 2.3. Measurement of pH

After preparing the milk samples, the pH was measured in duplicate, using a pH meter (VWR pH 1100L, Radnor, PA, USA). The pH meter was calibrated using technical buffer solutions of pH 4 and pH 7 (VWR, Radnor, PA, USA) before measurement. 

### 2.4. Proximate Analysis

#### 2.4.1. Moisture and Ash Content

The moisture content was determined following the method described by Ekanem [25]. The ash content was determined by heating the freeze-dried samples in an ash oven (Carbolite Furnaces CSF 1100, Aartselaar, Belgium) at 550 °C for 8 h, after the method of Ekanem [27]. Afterward the ash content was calculated by using the following equation:

Ash content % = (weight of the sample after ash oven)/(weight of the sample before ash oven) × 100%

#### 2.4.2. Fat Content 

The fat content was determined by the method of Manirakiza et al. [28], with the following modifications: one gram of freeze-dried sample was weighed on an analytical balance (Mettler-Toledo XA105, Giessen, Germany) with an accuracy of 0.1 mg in extraction thimbles with 200 mL petroleum ether (Merck 1.01774.2500, Burlington, MA, USA) in flat-bottom flasks. The flat-bottom flask was connected to the extractor, put on a heating block (Labotech blocks, Gottingen, Germany), and boiled for at least 3 h. After the extraction, the petroleum ether in the flat-bottom flask was left to cool for 1 h and evaporated using a rotary evaporator (Büchi Rotavapor R-300, Essen, Germany) under vacuum. The flat-bottom flask was then left overnight to get rid of the last bit of petroleum ether and then weighed on the same analytical balance used at the beginning of the experiment.

#### 2.4.3. Protein Content 

Protein content was analyzed in duplicate using a LECO CN 628 Dumas analyzer, Bonn, Germany. Powdered treatment samples and blank samples with cellulose (Sigma Aldrich 310697, St. Louis, MO, USA) were weighed in tin cups (Interscience, Ottignies-Louvain-la-Neuve, Belgium) and put in a sample tray with 40 positions covered with aluminum foil to protect the tin cups. The samples were then oxidized in the Dumas metal column filled with chemicals at high temperature with water filters in a separation column on which nitrogen was separated and calculated into protein content. The conversion factor used to calculate the protein content was 6.25 for cow milk protein and 5.8 for soya milk protein [29].

### 2.5. Mineral Content 

The mineral content was measured by digesting the samples in a microwave oven (MARS 6 iWave, CEM Corporation, Mattews, NC, United States of Americawith sulfuric acid, hydrogen peroxide, and hydrochloric acid [30]. This solution was atomized in argon plasma, and due to the high temperature, the sample dried further and was ashed, atomized, and ionized. The minerals were detected using an ICP-MS (Agilent 7800 ICP MS, Matthews, NC, USA), and the signal intensity of the mineral was divided by the charge of the minerals [31]. The mineral content was quantified by means of a calibration line based on the ratio between the signal of the element and the signal of the associated standard.

### 2.6. Total Phenol Content

The total phenol content was determined by the Folin–Ciocalteu method [32], with the following modifications: the extraction of the phenolic compounds in turmeric-fortified milk samples was done by weighing 0.5 g of freeze-dried material in a 15 mL disposable tube. After that, 4 mL preheated CH_3_OH 100% (Merck, Burlington, MA, USA) incubated at 75 °C for 60 min (Julabo sw-20C, Seelbach, Germany) was added to the disposable tube and further incubated for 20 min at 75 °C. The tubes were mixed every 5 min using a vortex (Scientific Industries Vortex Genie 1, Bohemia, New York, NY, USA). After 20 min, the tubes were centrifuged (Eppendorf AG Centrifuge 5430 R, Harmburg, Germany) at 2500 rpm (699 g) for 10 min at 20 °C. The supernatant was collected, and the pellet was re-extracted two times, with 2 mL 70% CH_3_OH at 20 min incubation time. The supernatants were combined and stored in the freezer at −20 °C until further analysis. The supernatant (1 mL), 5 mL of demi water, 1 mL Folin–Ciocalteu reagents (Merck 109001.0500, Burlington, MA, USA) and 1 mL of saturated Na_2_CO_3_ (Merck 6329, USA) were added into a 25 mL volumetric flask. The volume was adjusted to 25 mL with demi water and mixed well by hand. The absorbance was measured at 750 nm using a spectrophotometer (Cary 50 Bio UV–Vis spectrophotometer, Walnut Creek, CA, USA) after 15 min. The gallic acid equivalent (GAE) was calculated using gallic acid (Sigma Aldrich 27654, St. Louis, MO, USA) as a reference standard. The concentration range of the gallic acid standard curve was 0.02–0.5 mg/mL. The standard curve was linear, and the equation of the line used was y = 4.3008x + 0.0219, with R^2^ = 0.9998.

### 2.7. Total Antioxidant Activity 

The total antioxidant activity was measured by the Quencher procedure [33] with the following modifications: a stock solution was made with 1,1-diphenyl-2-picryl-hydrazyl (DPPH; Sigma Aldrich, St. Louis, MO, USA) by preparing 10 mg DPPH/mL 100% pure ethanol (Merck, Burlington, MA, USA). The stock solution was then diluted to 5 mg/mL in Milli-Q water (VWR, Radnor, PA, USA) and subsequently diluted 125× by adding 200 µL DPPH stock to 25 mL 50% ethanol. This solution was prepared 24 h in advance in the dark to activate it. The calibration curve was prepared with 6 hydroxy 2,5,7,8 tetramethylchroman 2-carboxylic acid (Trolox 23881-3; Sigma Aldrich, St. Louis, MO, USA). A stock solution of Trolox in 100% ethanol was prepared by adding 20 mg of Trolox to 1 mL of 100% ethanol. This stock solution was diluted to 5.33×, 8×, 16×, and 40× in 100% ethanol to get four points calibration curve of 3.75, 2.5, 1.25, and 0.50 mg/mL. In addition, a blank of 100% ethanol were prepared in triplicates and the absorbance of the blank (average value of its triplicates) was taken as the initial concentration in the test (0.00 mmol). All freeze-dried milk samples were diluted with cellulose by combining 100 mg of cellulose with 100 mg of milk in an aluminum dish with a spatula (1:1 ratio). A 1:1 ratio was used for 0% turmeric in milk samples. The turmeric-fortified milk samples required a 2:1 ratio due to their high yellow pigmentation. Next, 10, 20, and 40 mg samples in 5 mL Eppendorf tubes were prepared, and 5 mL DPPH was added to each milk type and Trolox sample. After incubation in the Heidolph shaker (Multi Reax Vortexer, Schwabach, Germany), the samples were centrifuged for 5 min at 9000 rcf at room temperature. The absorbance of the supernatant was measured at 525 nm in the spectrophotometer (Cary 50 Bio UV–Vis spectrophotometer, Walnut Creek, CA, USA). 

### 2.8. Data Analysis

All variables are reported as means ± standard deviation (SD) with a minimum of two replicates. The Heatmapper web server was used to visualize all samples’ proximate and physicochemical composition [34]. The difference between samples with and without heat treatment was tested by two-way analysis of variance (ANOVA) followed by the post hoc Tukey test and one-way ANOVA to evaluate the relationship between variables and milk type using IBM SPSS Statistics 25. The differences in means were considered significantly different at *p* < 0.05.

## 3. Results and Discussion

### 3.1. Proximate and Physicochemical Composition

Before and after heat treatment, the moisture content for control samples ranged from 88.6–89.7%, 89.8–90.7%, and 88.4–89.5% for whole milk, skimmed milk, and soya milk, respectively. The moisture content of the turmeric-fortified whole milk, skimmed milk, and soya milk samples ranged from 86.7% to 88.5%, 90.4% to 91.3%, and 87.9% to 89.0%, respectively. For all the milk types, 6% turmeric-fortified samples had the lowest moisture content before and after heat treatments. However, the mean values of moisture contents of 2% turmeric-fortified samples were significantly lower than that of the control samples (*p* < 0.05). The same trends were observed between the 6% and 2% turmeric- fortified whole and soya milk. In addition, 6% heat-treated turmeric-fortified skimmed milk showed no significant difference from both 0% and 2% turmeric-fortified ones (*p* < 0.05). These results were similar to those of previous studies [35,36].

The ash content for control samples ranged from 0.7% to 0.8%, 1.0% to 1.1%, and 0.4% to 0.5% for whole milk, skimmed milk, and soya milk, respectively. The ash content of the turmeric-fortified skimmed milk ranged from 1.1% to 1.2%. The turmeric-fortified whole and soya milk ash contents were within the range of the control sample. Before heat treatment, the ash content of the turmeric-fortified samples differed significantly from the control samples for all the milk types. However, no significant differences were observed between the 2% and 6% turmeric-fortified samples (*p* < 0.05). After heat treatment, there were no significant differences observed among the control and fortified samples (*p* < 0.05). The fat content for both control and turmeric-fortified milk samples ranged from 0.1% to 1.6% with significant differences observed among all treatment for the turmeric-fortified whole and soya milk samples (*p* < 0.05). The pH of all samples was relatively stable and independent of turmeric addition before and after heat treatment.

**Figure 3:**
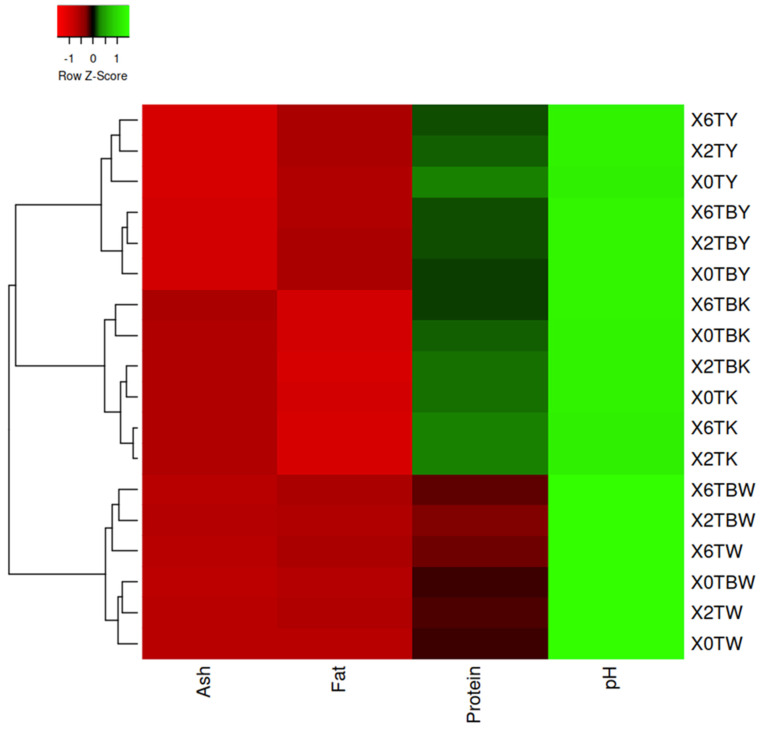
Heatmap of the pH, ash, fat, and protein content of three types of turmeric-fortified milk. Mean values refer to colors from minimum (displayed in red) to maximum (represented with green). Key: TW, TK, and TY represent turmeric-fortified whole milk, skimmed milk, and soya milk respectively. TBW, TBK, and TBY represent boiled turmeric-fortified whole milk, skimmed milk, and soya milk, respectively. X means sample and the number 0, 2, or 6 represents the percentage of turmeric in each sample.

The protein content for control samples ranged from 2.3% to 2.4%, 3.4% to 3.6%, and 3.5% to 4.0% for whole milk, skimmed milk, and soya milk, respectively. The protein content of the turmeric-fortified whole milk, skimmed milk, and soya milk samples ranged from 1.7% to 2.1%, 3.1% to 3.8%, and 3.5% to3.9%, respectively. Natural crude polyphenols from plants such as grape (*Vitis vinifera*) and tea (*Camellia sinensis*), and cranberry (*Vaccinium oxycoccos*) extracts have been reported to have strong bindings with milk proteins [37]. Thus, the reduction in the protein contents for the milk samples fortified with turmeric could be because of the binding by polyphenols originating from the turmeric, though, the reduction showed no significant different at (*p* < 0.05). Overall, the results in this study were in line with the proximate composition of herbal *sandesh*, an Indian coagulated milk product with turmeric, spinach (*Spinacia oleracea*), curry leaf (*Murraya koenigii*), coriander (*Coriandrum sativum*), and aonla (*Emblica officinalis*) [38]. The proximate and physicochemical compositions visualized with the heatmap (Figure 3) show that the samples fall into three clusters according to milk type. 

### 3.2. Mineral Content

Zinc and iron are the most predominant deficient micronutrients in human diets globally [39]. Calcium is also an important mineral found in milk. Consumers may be at risk of calcium deficiencies if they substitute non-fortified and non-supplemented plant drinks for cow’s milk [40]. Thus, the minerals measured in this present study were calcium, iron, and zinc. The calcium, iron, and zinc contents of turmeric-fortified whole and soya milk samples after heat treatment ranged from 17.8 to 85.1, 0.37 to 0.53, and 0.29 to 0.30 mg/100 mL, respectively. A calcium content of 120 mg/100 mL was declared on the packaging of both the whole and soya milk used in this study. This value was high compared to the calcium content of our control samples, with soya milk showing the lowest values. Soya milk does not naturally contain such high levels of calcium as declared on the packaging. However, soya milk is generally fortified with calcium to create a comparable composition to cow milk to make it a suitable milk alternative [41]. The higher the storage temperature, the higher the sedimentation in milk beverages [42]. The soya milk in this research was stored at room temperature, which may have caused high sedimentation in the package. We suspect that this could have influenced the mineral composition of the control soya milk samples.

Nevertheless, the amount of calcium as well as iron and zinc in all soya milk samples in this study agrees with [43], who reported values of 18.5, 0.5, and 0.3 mg/100 mL for calcium, iron, and zinc, respectively, for freshly prepared soya milk. Both iron and zinc increase with increasing concentration of turmeric. The values of these minerals in samples with 6% turmeric agreed with previous research [43]. The value was higher than commonly reported in the literature [8,44]. The differences in the absorption of iron result not only from individual variability but also from the type of food in which it is supplied. Iron of milk origin is absorbed much better than the iron from turmeric. Furthermore, the ash content, which represents the total minerals in food [45], did not increase with the addition of turmeric in the turmeric-fortified soya milk samples. Thus, turmeric only seems to prevent loss of minerals due to sedimentation in soya milk during storage.

### 3.3. Total Phenol Content 

Milk type, turmeric paste concentration, and heat treatment (Table 1) had a significant effect (*p* < 0.05) on the total phenol content (TPC) of golden milk. The TPC value of control whole milk was lower than the values reported previously [32,46]. It should be noted though that the milk used in this study was commercially available, UHT-sterilized milk. The severe thermal sterilization treatment might have triggered reactions that affected the phenolic composition by releasing the phenolic compounds from their bonded forms, causing subsequent degradation, hydrolysis, and transformations [47]. Moreover, even though commercial milk samples were used by others, the freeze-drying process to obtain homogenous and solid samples, this could have lowered the TPC in our study [46].

Turmeric-fortified soya milk showed a higher phenol content than turmeric-fortified cow milk, which agrees with previous research [48]. Without turmeric, the TPC of soya milk was 0.09 GAE/kg sample. The TPC of soya milk was higher than that of whole milk and skimmed milk. Moreover, 2% turmeric gave 0.10 GAE/kg and 6% turmeric, 0.14 GAE/kg. Soya beans are a good source of many nutrients, including polyphenols. Values of TPC in soya bean depend on variety and practices to produce soya milk, causing variations between products and brands [49].

Adding turmeric generally showed a significant increase in the TPC for the three milk types. Adding a higher amount of turmeric (6%) is beneficial from a nutrition perspective [50]. Even though the TPC decreased after heat treatment when 6% turmeric was added to all the milk samples, boiling milk protects the milk from spoilage microorganisms. Considering the challenges of improving hygiene in developing countries, boiling milk at low–medium heat is strongly recommended [51]. In general, however, the boiling (which would be good from a food safety perspective) does not annihilate the increase of TPC due to the 2% addition of turmeric. Besides, whilst 0% turmeric in boiled skimmed milk showed the lowest TPC, the addition of turmeric complemented this deficit with 6% turmeric in boiled skimmed milk, which roughly showed a 25% increase.

### 3.4. Antioxidant Activity

The addition of turmeric showed a significant increase in antioxidant activity, and when more turmeric was added to whole milk, the antioxidant activity increased (Table 2). Heat treatment of whole milk showed a significant decrease for the samples with 0% and 2% turmeric but a slight increase for the sample with 6% turmeric. Overall, the antioxidant activity increased with the addition of turmeric. Whole milk before treatment showed a similar value of the antioxidant activity as skimmed milk. These values differed from the value reported in the literature [52]. The low fat content of our whole milk sample may be responsible for the discrepancy as the antioxidant activity has been linked to the chemical content of milk from different species, particularly in cows [53]. The difference could also be due to different processing methods applied before measurement. According to Khan et al. [16], boiling did not affect the antioxidant activity of cow milk, which is in line with the whole milk samples in this research.

The antioxidant activity of skimmed milk without turmeric was low, and the addition of turmeric increased the antioxidant activity significantly. Heat treatment did not significantly affect the antioxidant activity of the skimmed milk samples. In line with previous research [54], neither the differences in fat content nor heat treatment caused significant differences in the antioxidant values. Raw milk, which is commonly used in studies to measure antioxidant activity, has different properties than the commercial UHT milk used in the present research [55].

The initial antioxidant activity of soya milk without turmeric was much higher than that of whole milk and skimmed milk. The addition of 2% and 6% turmeric showed a large increase in antioxidant activity. Heat treatment also significantly increased the antioxidant activity. The significantly higher antioxidant activity of soya milk compared to that of cow milk agrees with previous research [56]. Soya bean is known to have a high antioxidant activity due to polyphenolic antioxidants, such as isoflavones. Overall, the addition of turmeric to both cow and soya milk formed a nutritionally better product [57]. Furthermore, in this research, soya milk showed the highest antioxidant activity, regardless of heat treatment.

## 4. Conclusions

Consumer preferences for healthy foods has increased the popularity of golden milk across the globe. To the best of our knowledge, this is the first scientific literature to investigate the antioxidant and nutritional compositions of this widely consumed functional drink. This study also provides a basis for developing novel turmeric-fortified soya milk, which is nutritionally equivalent to the popular golden milk (turmeric-fortified cow milk). Turmeric added nutritional and chemical values to all three milk varieties without heat treatment. The effect of heat treatment on turmeric-fortified milk significantly impacted the TPC of 0% and 6% turmeric in whole milk. The phenolic compounds, antioxidant activity, protein, and iron increased with 2% and 6% turmeric in soya milk. Thus, soya milk appears to be the best matrix to deliver the antioxidant benefits of turmeric to the cow-milk-allergic populace and the low-income population of urbanized areas in developing countries. Overall, this research serves as a starting point to validate the claims of golden milk’s benefits. The present work standardized the levels of turmeric concentration and also studied the effect of heat treatment using soya milk as alternative. However, further research is recommended to study the bioavailability of minerals and bioactive compounds after the intake of turmeric-fortified milk.

## Figures and Tables

**Table 1:** Total phenolic compounds, expressed in milligrams of GAE per kg of whole milk, skimmed milk, and soya milk.

		GAE (g/kg Sample)	
	Sample	Unboiled	Boiled
Whole milk	0% Turmeric	0.01 ± 0.5 ^a,1^	0.03 ± 0.2 ^a,1^
	2% Turmeric	0.03 ± 0.2 ^a,2^	0.04 ± 0.2 ^a,2^
	6% Turmeric	0.05 ± 0.4 ^a,3^	0.05 ± 1.6 ^a,2^
Skimmed milk	0% Turmeric	0.01 ± 0.1 ^a,1^	0.02 ± 0.6 ^a,1^
	2% Turmeric	0.04 ± 0.9 ^a,2^	0.05 ± 2.2 ^a,1^
	6% Turmeric	0.08± 1.5 ^b,3^	0.08 ± 1.2 ^a,1^
Soya milk	0% Turmeric	0.09 ± 0.2 ^b,1^	0.10 ± 1.7 ^b,1^
	2% Turmeric	0.10 ± 1.1 ^b,1^	0.11 ± 0.7 ^b,2^
	6% Turmeric	0.14 ± 1.6 ^c,2^	0.13 ± 2.2 ^b,3^

Values are the means of three independent measurements. ^a–c^ Different letters indicate a significant difference between the three types of turmeric-fortified milk with the same turmeric percentage and heat treatment. ^1–3^ Different numbers indicate a significant difference for a certain turmeric percentage within the same milk type and heat treatment (*p* < 0.05).

**Table 2:** Antioxidant activities of turmeric-fortified whole milk, skimmed milk, and soya milk before and after heat treatment expressed in Trolox equivalent antioxidant capacity (TEAC).

		TEAC (mmol Trolox/kg Sample)	
	Sample	Unboiled	Boiled
Whole milk	0% Turmeric	0.5± 0.1 ^a,1^	0.1 ± 0.1 ^a,1^
	2% Turmeric	3.8 ± 0.4 ^ab,2^	1.4 ± 0.1 ^a,2^
	6% Turmeric	5.1 ± 1.1 ^a,2^	5.3 ± 0.1 ^a,3^
Skimmed milk	0% Turmeric	0.5 ± 0.4 ^a,1^	0.4 ± 0.2 ^b,1^
	2% Turmeric	2.9 ± 0.3 ^a,2^	2.6 ± 0.4 ^a,2^
	6% Turmeric	5.6 ± 0.5 ^a,3^	5.6 ± 0.1 ^a,3^
Soymilk	0% Turmeric	3.4 ± 0.1 ^b,1^	7.5 ± 0.3 ^a,1^
	2% Turmeric	4.6 ± 0.2 ^b,1^	11.6 ± 0.7 ^b,2^
	6% Turmeric	4.5 ± 0.8 ^a,1^	17.7 ± 0.9 ^b,3^

Different letters indicate a significant difference in milk type within the same turmeric percentage and heat treatment. Different numbers indicate a significant difference in turmeric percentage within the same milk type and heat treatment (*p* < 0.05).

## Data Availability

Not applicable.

## References

[1] Nwaekpe J., Anyaegbunam H., Okoye B., Asumugha G. (2015). Promotion of turmeric for the food/pharmaceutical industry in Nigeria. J. Exp. Agric. Int..

[2] Hewlings S.J., Kalman D.S. (2017). Curcumin: A review of its effects on human health. Foods.

[3] Idowu-Adebayo F., Fogliano V., Oluwamukomi M.O., Oladimeji S., Linnemann A.R. (2021). Food neophobia among Nigerian consumers: A study on attitudes towards novel turmeric-fortified drinks. J. Sci. Food Agric..

[4] Idowu-Adebayo F., Toohey M.J., Fogliano V., Linnemann A.R. (2021). Enriching street-vended zobo (*Hibiscus sabdariffa*) drink with turmeric (*Curcuma longa*) to increase its health-supporting properties. Food Funct..

[5] Araujo C., Leon L. (2001). Biological activities of *Curcuma longa* L.. Memórias Inst. Oswaldo Cruz.

[6] Velayudhan K., Dikshit N., Nizar M.A. (2012). Ethnobotany of turmeric (*Curcuma longa* L.). Indian J. Tradit. Knowl..

[7] Čosić A., Karić A., Šabanović K., Šutković J., Yildirim A. (2020). Determination of GMO soy products in processed food from Bosnian market. Bioeng. Stud..

[8] Mazumder M.A.R., Begum A.A. (2016). Soy milk as source of nutrient for malnourished population of developing country: A review. Int. J. Adv. Sci. Tech. Res..

[9] Ezeigbo O., Ekaiko M., Kalu T., Nwodu J. (2014). Quality Assessment of Soymilk Sold in Aba, Southeastern Nigeria. Int. J. Epidemiol. Infect..

[10] Osuntogun B., Aboaba O. (2004). Microbiological and physico-chemical evaluation of some non-alcoholic beverages. Pak. J. Nutr..

[11] Sęczyk Ł., Świeca M., Gawlik-Dziki U. (2017). Soymilk enriched with green coffee phenolics–Antioxidant and nutritional properties in the light of phenolics-food matrix interactions. Food Chem..

[12] Paul A.A., Kumar S., Kumar V., Sharma R. (2020). Milk Analog: Plant based alternatives to conventional milk, production, potential and health concerns. Crit. Rev. Food Sci. Nutr..

[13] Silván J.M., Amigo-Benavent M., del Castillo M.D. (2014). Antioxidant Properties of Soy-Based Drinks and Effects of Processing. Processing and Impact on Antioxidants in Beverages.

[14] Rizzo G., Baroni L. (2018). Soy foods and their role in vegetarian diets. Nutrients.

[15] Vanderhout S.M., Aglipay M., Torabi N., Jüni P., da Costa B.R., Birken C.S., O’Connor D.L., Thorpe K.E., Maguire J.L. (2020). Whole milk compared with reduced-fat milk and childhood overweight: A systematic review and meta-analysis. Am. J. Clin. Nutr..

[16] Khan I.T., Nadeem M., Imran M., Ayaz M., Ajmal M., Ellahi M.Y., Khalique A. (2017). Antioxidant capacity and fatty acids characterization of heat treated cow and buffalo milk. Lipids Health Dis..

[17] Jung S., Murphy P.A., Sala I. (2008). Isoflavone profiles of soymilk as affected by high-pressure treatments of soymilk and soybeans. Food Chem..

[18] Ren C., Xiong W., Peng D., He Y., Zhou P., Li J., Li B. (2018). Effects of thermal sterilization on soy protein isolate/polyphenol complexes: Aspects of structure, in vitro digestibility and antioxidant activity. Food Res. Int..

[19] Ozdal T., Capanoglu E., Altay F. (2013). A review on protein–phenolic interactions and associated changes. Food Res. Int..

[20] El-Sayed S.M., Youssef A.M. (2019). Potential application of herbs and spices and their effects in functional dairy products. Heliyon.

[21] Bhattacharyya S., Chakraborty C., Moitra S., Bandyopadhyay K. (2017). Potential application of milk and milk products as carrier for herbs and spices: A Review. Int. J. Eng. Res. Sci. Technol..

[22] Yamamoto N., Shoji M., Hoshigami H., Watanabe K., Takatsuzu T., Yasuda S., Igoshi K., Kinoshita H. (2019). Antioxidant capacity of soymilk yogurt and exopolysaccharides produced by lactic acid bacteria. Biosci. Microbiota Food Health.

[23] Pratap D., Singh R., Ravichandran C., Ojha A., Upadhyay A., Kaur B.P., Senthilkumar T. (2019). Evaluation of physicochemical, antioxidant, and sensory characteristics of khoa prepared from blends of soy and standardized milk. J. Food Process. Preserv..

[24] Fardet A., Rock E. (2018). In vitro and in vivo antioxidant potential of milks, yoghurts, fermented milks and cheeses: A narrative review of evidence. Nutr. Res. Rev..

[25] Park H., Lee M., Kim K.-T., Park E., Paik H.-D. (2018). Antioxidant and antigenotoxic effect of dairy products supplemented with red ginseng extract. J. Dairy Sci..

[26] Ye J., Fan F., Xu X., Liang Y. (2013). Interactions of black and green tea polyphenols with whole milk. Food Res. Int..

[27] Ekanem J.O. (2018). Microbial, sensory and nutritional properties of laboratory prepared sorrel (zobo) drinks fortified with spices and sugar. J. Glob. Biosci..

[28] Manirakiza P., Covaci A., Schepens P. (2001). Comparative study on total lipid determination using Soxhlet, Roese-Gottlieb, Bligh & Dyer, and modified Bligh & Dyer extraction methods. J. Food Compos. Anal..

[29] Sáyago-Ayerdi S.G., Arranz S., Serrano J., Goñi I. (2007). Dietary fiber content and associated antioxidant compounds in roselle flower (*Hibiscus sabdariffa* L.) beverage. J. Agric. Food Chem..

[30] Meyer S., Markova M., Pohl G., Marschall T.A., Pivovarova O., Pfeiffer A.F.H., Schwerdtle T. (2018). Development, validation and application of an ICP-MS/MS method to quantify minerals and (ultra-)trace elements in human serum. J. Trace Elem. Med. Biol..

[31] Temminghoff E., Houba V. (2004). Plant Analyses Procedures.

[32] Vázquez C.V., Rojas M.G.V., Ramírez C.A., Chávez-Servín J.L., García-Gasca T., Martínez R.A.F., García O.P., Rosado J.L., López-Sabater C.M., Castellote A.I. (2015). Total phenolic compounds in milk from different species. Design of an extraction technique for quantification using the Folin–Ciocalteu method. Food Chem..

[33] Serpen A., Gökmen V., Fogliano V. (2012). Solvent effects on total antioxidant capacity of foods measured by direct Quencher procedure. J. Food Compos. Anal..

[34] Benabderrahim M.A., Yahia Y., Bettaieb I., Elfalleh W., Nagaz K. (2019). Antioxidant activity and phenolic profile of a collection of medicinal plants from Tunisian arid and Saharan regions. Ind. Crops Prod..

[35] Hajirostamloo B., Mahastie P. (2009). Comparison of nutritional and chemical parameters of soymilk and cow milk. World Acad. Sci. Eng. Technol..

[36] Woodside J.V., Brennan S., Cantwell M. (2016). Are Soy-Milk Products Viable Alternatives to Cow’s Milk?. Beverage Impacts on Health and Nutrition.

[37] Han J., Chang Y., Britten M., St-Gelais D., Champagne C.P., Fustier P., Lacroix M. (2019). Interactions of phenolic compounds with milk proteins. Eur. Food Res. Technol..

[38] Bandyopadhyay M., Chakraborty R., Raychaudhuri U. (2007). Incorporation of herbs into sandesh, an Indian sweet dairy product, as a source of natural antioxidants. Int. J. Dairy Technol..

[39] Kihara J., Bolo P., Kinyua M., Rurinda J., Piikki K. (2020). Micronutrient deficiencies in African soils and the human nutritional nexus: Opportunities with staple crops. Environ. Geochem. Health.

[40] Scholz-Ahrens K.E., Ahrens F., Barth C.A. (2020). Nutritional and health attributes of milk and milk imitations. Eur. J. Nutr..

[41] Nieves J.W. (2005). Osteoporosis: The role of micronutrients. Am. J. Clin. Nutr..

[42] Koffi E., Shewfelt R., Wicker L. (2005). Storage stability and sensory analysis of uht-processed whey-banana beverages. J. Food Qual..

[43] Nti C.A., Plahar W.A., Annan N.T. (2016). Development and quality characteristics of shelf-stable soy-agushie: A residual by-product of soymilk production. Food Sci. Nutr..

[44] Alozie Yetunde E., Udofia U.S. (2015). Nutritional and sensory properties of almond (*Prunus amygdalu Var. Dulcis*) seed milk. World J. Dairy Food Sci..

[45] Marshall M.R. (2010). Ash analysis. Food analysis.

[46] Ertan K., Bayana D., Gokce O., Alatossava J.T., Yilmaz Y., Gursoy O. (2017). Total antioxidant capacity and phenolic content of pasteurized and UHT-treated cow milk samples marketed in Turkey. Acad. Food J..

[47] Chávez-Servín J.L., Andrade-Montemayor H.M., Vázquez C.V., Barreyro A.A., García-Gasca T., Martínez R.A.F., Ramírez A.M.O., de la Torre-Carbot K. (2018). Effects of feeding system, heat treatment and season on phenolic compounds and antioxidant capacity in goat milk, whey and cheese. Small Rumin. Res..

[48] Apostolidis E., Kwon Y.-I., Shinde R., Ghaedian R., Shetty K. (2011). Inhibition of Helicobacter pylori by fermented milk and soymilk using select lactic acid bacteria and link to enrichment of lactic acid and phenolic content. Food Biotechnol..

[49] McClements D.J., Newman E., McClements I.F. (2019). Plant-based milks: A review of the science underpinning their design, fabrication, and performance. Compr. Rev. Food Sci. Food Saf..

[50] Ogori A.F., Amove J., Aduloju P., Sardo G., Okpala C.O.R., Bono G., Korzeniowska M. (2021). Functional and Quality Characteristics of Ginger, Pineapple, and Turmeric Juice Mix as Influenced by Blend Variations. Foods.

[51] Amenu K., Agga G.E., Kumbe A., Shibiru A., Desta H., Tiki W., Dego O.K., Wieland B., Grace D., Alonso S. (2020). Milk Symposium review: Community-tailored training to improve the knowledge, attitudes, and practices of women regarding hygienic milk production and handling in Borana pastoral area of southern Ethiopia. J. Dairy Sci..

[52] Niero G., Penasa M., Currò S., Masi A., Trentin A., Cassandro M., De Marchi M. (2017). Development and validation of a near infrared spectrophotometric method to determine total antioxidant activity of milk. Food Chem..

[53] Niero G., Currò S., Costa A., Penasa M., Cassandro M., Boselli C., Giangolini G., De Marchi M. (2018). Phenotypic characterization of total antioxidant activity of buffalo, goat, and sheep milk. J. Dairy Sci..

[54] Michlova T., Dragounova H., Horníčková Š., Hejtmankova A. (2015). Factors influencing the content of vitamins A and E in sheep and goat milk. Czech J. Food Sci..

[55] Yilmaz-Ersan L., Ozcan T., Akpinar-Bayizit A., Sahin S. (2018). Comparison of antioxidant capacity of cow and ewe milk kefirs. J. Dairy Sci..

[56] Baghbadorani S.T., Ehsani M.R., Mirlohi M., Ezzatpanah H., Azadbakht L., Babashahi M. (2017). Antioxidant capability of ultra-high temperature milk and ultra-high temperature soy milk and their fermented products determined by four distinct spectrophotometric methods. Adv. Biomed. Res..

[57] Naik L., Ankitha R., Sharma A. (2018). Salubrious Curcumin Fortified Whey Beverage Formulation and Study its Antioxidant Property. Int. J. Sci. Eng. Manag..

